# The Association of Oxidative Stress in the Uvular Mucosa with Obstructive Sleep Apnea Syndrome: A Clinical Study

**DOI:** 10.3390/jcm10051132

**Published:** 2021-03-08

**Authors:** Ewa Olszewska, Joanna Rogalska, Małgorzata M. Brzóska

**Affiliations:** 1 Department of Otolaryngology, Medical University of Bialystok, 15-089 Bialystok, Poland; 2 Department of Toxicology, Medical University of Bialystok, 15-089 Bialystok, Poland; joanna.rogalska@umb.edu.pl (J.R.); malgorzata.brzoska@umb.edu.pl (M.M.B.)

**Keywords:** obstructive sleep apnea, oxidative/reductive status, oxidative stress, sleep surgery, uvular mucosa

## Abstract

The hypothesis that individuals with obstructive sleep apnea syndrome (OSAS) demonstrate oxidative stress in the uvular mucosa that correlates with OSAS occurrence was investigated. A total of 128 participants (mean age 45.8, mean body mass index 30.7, female–male ratio 1:20) were divided into the non-OSAS group (apnea–hypopnea index—AHI < 5) and OSAS-group (AHI ≥ 5), in which mild (5 ≤ AHI < 15), moderate (15 ≤ AHI < 30), and severe (AHI ≥ 30) sub-groups were distinguished. Laryngological examination, Epworth Sleep Scale questionnaire, and home sleep study were performed to obtain AHI, mean oxygen saturation, and lowest oxygen saturation. Total oxidative status (TOS) and total antioxidative status (TAS) were assayed in the uvular mucosa taken during palatoplasty or palatopharyngoplasty. The severity of oxidative stress was expressed as oxidative stress index (OSI). Oxidative/reductive imbalance was noted in the mucosa of the uvula of OSAS individuals, and TAS of the uvular mucosa negatively correlated with the severity of this syndrome. TOS and OSI in the mild, moderate, and severe OSAS were higher than in the non-OSAS group, whereas TAS of the uvular mucosa in the OSAS group was lower compared to the non-OSAS group. In conclusion, oxidative stress in the uvular mucosa is associated with the occurrence of OSAS.

## 1. Introduction

Sleep-disordered breathing (SDB) is a series of disorders including snoring and obstructive sleep apneas. Obstructive sleep apnea syndrome (OSAS) is a nocturnal disorder characterized by recurrent episodes of upper airway obstruction during sleep, associated with oxygen desaturation and sleep fragmentation [1,2]. The severity of OSAS is measured by the apnea–hypopnea index (AHI), which is defined by the number of apneas and hypopneas events per hour of sleep. Episodes of apneas and hypopneas during sleep that last a minimum of 10 s are usually followed by a significant decrease in arterial oxygen saturation. It induces excessive daytime sleepiness, daytime fatigue, disturbance of concentration, deficits in cognitive complaints, and mood disorders that lead to an increased risk for traffic accidents and accidents during work, as well as an increased number of days off work [2].

The pathogenesis of OSAS is multifactorial and still not fully established. The disease is highly associated with higher body mass index (BMI), male gender, and older age [3,4,5,6]. It involves a diverse range of mechanisms including selective activation of inflammatory molecular pathways, endothelial dysfunction, metabolic dysregulation, and oxidative stress [7,8]. Endothelial dysfunction is often considered as one of the earliest detectable and possibly reversible abnormalities during the development of atherosclerosis [9]. Studies indicate an association between the presence of both coronary and systemic endothelial dysfunction and an increased risk of future cardiovascular morbidity and mortality in patients with OSAS [10]. Consequently, the severity of endothelial dysfunction may depend on the severity of OSAS. Moreover, the development of cardiovascular morbidity and mortality may also occur secondary to other pathologies caused by OSAS, such as hypertension and diabetes [11]. OSAS was found to act as a contributor to the pathogenesis of cardiovascular diseases. It is associated with a risk of hypertension (resistant hypertension and pulmonary one), heart failure, arrhythmias (e.g., atrial fibrillation), coronary heart disease, cardiomyopathy, and stroke [12,13,14]. 

The mechanisms of tissue injury in OSAS are not completely known. In intermittent hypoxia during sleep, the inflammatory signaling factors play essential roles in the transcriptional regulation of inflammatory cytokines and stimulate oxidative stress. Oxidative stress is caused by an imbalance between the oxidant-producing systems (production of reactive oxygen species—ROS) and antioxidant defense mechanisms. It results in the activation of redox-sensitive transcription factors. Some of them such as monocyte chemotactic protein 1 (MCP-1), interleukin-6, and the excessive formation of ROS might lead to more severe OSAS [15,16]. On the other hand, worsened OSAS may then be further increasing the levels of ROS, leading a vicious cycle. ROS can damage various cellular and molecular components and plays a role in the activation of multiple genes [17]. To date, we are not aware of any studies that have evaluated the oxidative/antioxidative status in tissues of sleep apnea patients.

We hypothesized that the oxidative/reductive status in individuals with OSAS is different than in the non-OSAS group and correlates with the severity of OSAS. To investigate this hypothesis, we evaluated the oxidative stress parameters in the enlarged mucosa of the uvula of participants who underwent a surgical procedure due to OSAS and compared them with the non-OSAS group. For this purpose, we evaluated disturbances in the oxidative/antioxidative balance in the mucosa of the uvula on the basis of the measurement of total oxidative status (TOS) and total antioxidative status (TAS) and calculation of oxidative stress index (OSI = TOS/TAS), which reflects the severity of oxidative stress. The main aims of the study included the evaluation of whether the imbalance in the oxidative/antioxidative status in the mucosa of the uvula occurs in the participants with OSAS and the assessment of the relationship between the extent of oxidative stress in the mucosa of the uvula and the severity of OSAS. Revealing the existence of dependence between the oxidative/reductive status of the uvular mucosa and the severity of OSAS will provide insight into these pathways (mechanisms and causes). This insight may enhance the understanding of the relationship between OSAS and oxidative stress and show the reasonableness to administer adjuvant treatment, such as antioxidants.

## 2. Materials and Methods

### 2.1. Study Protocol

The study was approved by the Bioethics Committee of the Medical University of Bialystok (Poland; approval no. R-I-002/535/2017) and conducted according to GCP/Guidelines for Good Clinical Practice. A signed written informed consent form and acceptance to participate in the study, as well as acceptance to have the uvular mucosa evaluated, were received from all participants of the study.

The study was performed among the patients treated at the Department of Otolaryngology in a tertiary care hospital with primary snoring and OSAS, enrolled on the basis of inclusion and exclusion criteria. All patients underwent the same procedures according to the study protocol: a medical history, Epworth Sleep Scale (ESS) questionnaire, visual analog scale for snoring loudness, BMI, endoscopy of the upper airways, polygraphy (sleep study type III), and a surgical procedure. The ESS is a self-administered questionnaire developed by Murray Johns, consisting of 8 questions about the possibilities of falling asleep in different situations such as sitting and reading, watching TV, sitting inactive in a public place, riding as a passenger in a car for an hour without a break, lying down to rest in the afternoon when circumstances permit, sitting and talking to someone, sitting quietly after a lunch without alcohol, and sitting in a car that has stopped for a few minutes in traffic [18]. The patient rates these possibilities on a scale of 0 to 3 (0—never doze, 3—high chance of dozing). The total score can range from 0 to 24. The normal range of sleepiness in healthy adults varies from 0 to 10. A higher score is associated with increased sleepiness [19]. Snoring was primarily evaluated by the patient with a 10 cm visual analogue scale (VAS) at baseline. The participants, on the basis of descriptions from their spouse or bed partner, were asked to estimate the severity of their snoring using a 10 cm VAS from 0 (no snoring) to 10 (very severe snoring, bed partner leaves the room).

#### 2.1.1. Inclusion and Exclusion Criteria

Inclusion criteria to the OSAS group contained patients over 18 years with OSAS confirmed on the basis of a home sleep study type III (polygraphy). The non-OSAS group consisted of adult patients suffering from primary snoring but not from OSAS, defined as AHI < 5 in polygraphy. They did not suffer from any chronic disease or immune disease and did not receive any treatment for snoring 6 months before the enrollment in the study.

We excluded patients from either the non-OSAS or OSAS groups in a case of central sleep apnea syndrome; severe obesity (BMI ≥ 35); drug and alcohol abuse; smokers above 5 cigarettes per day; a treatment for sleep apnea within the 3 months before enrollment; a history of respiratory infection within the previous 4 weeks; a history of rheumatic diseases, coagulation disorders, or acute/chronic kidney failure (renal failure defined as serum creatinine > 2.0 mg/dL); a history of injury or surgery in the past 3 months; presence of other respiratory diseases such as asthma or chronic obstructive pulmonary disease with regard to clinical history or chest radiography; cardiovascular diseases; systemic inflammatory diseases; diabetes; comorbidities that may affect systemic inflammation such as collagen vascular disease or cancer; chronic rhinosinusitis; and receiving medical treatment such as hormones, immune suppressors, cytotoxins, or free radical scavengers. All cardiovascular diseases were considered as exclusion criteria, e.g., abnormal heart rhythms or arrhythmias, aorta disease and Marfan syndrome, congenital heart disease, coronary artery disease (narrowing of the arteries), deep vein thrombosis and pulmonary embolism, heart attack, heart failure, heart muscle disease (cardiomyopathy), heart valve disease, pericardial disease, peripheral vascular disease, rheumatic heart disease, stroke, and vascular disease (blood vessel disease). Additionally, an excessive fat accumulation in the neck triggers the release of pro-inflammatory cytokines, leading to the inflammatory response that may disturb the assessment of oxidative stress extent [20]. Moreover, severe obesity is contraindicated for surgical procedures both under local and general anesthesia [21].

After the diagnostic process, the possible therapeutic procedures were discussed with participants given a choice of non-surgical treatment for primary snoring or OSAS, such as diet, anti-snoring pillow, oral appliance, and positive airway pressure therapy.

#### 2.1.2. Sleep Study

The third type of sleep study was performed in each case using Alice Night One device, Philips Respironics. Each participant underwent a two-night home sleep study and was instructed on how to be ready for the study (avoid alcohol, sleeping pills before going to bed, avoid pajamas that are too tight). He/she was also instructed how and where to place sensors: effort belt (around the chest with Alice Night One device in the center of the chest), a nasal cannula (twisted clockwise to connect it to the top of the device, and each prong of the tube placed in each nostril), and pulse oximeter (placed on one of the index fingers). It measures oxygen saturation (SpO_2_, finger probe, Oximetry board Nonin), pulse rate (from the oximeter probe), airflow (pressure-based airflow with snore detection through a nasal cannula and thermistor), thoracic and abdominal effort (inductance plethysmography), and body position. The device includes an event button. The study was initiated individually with the start hour according to the participant’s night habits and the end time (6–8 h of recording). If there were less than 4 h of the recording, the data were not included in the analysis, and the repeated sleep study was obtained. During polygraphy, the following parameters were evaluated for this study: apnea–hypopnea index (AHI), mean oxygen saturation (MOS), lowest oxygen saturation (LOS), and oxygen desaturation index (ODI). There was a manual analysis performed for each sleep study.

AHI is described as the total number of apnea and hypopnea events per hour of sleep recorded in an overnight sleep study. Apnea is the condition of at least 90% decrease or complete cessation of airflow at the nose and mouth lasting at least 10 s during sleep whilst hypopnea is defined as at least 30% decrease in airflow at a nose and mouth with the oxygen desaturation of at least 3% lasting at least 10 s during sleep. MOS estimated as normal varies between 94% and 98% during sleep [2]. ODI is defined as the number of ≥3% arterial desaturations per hour of sleep [22].

All patients eligible to enroll in the study were dived into 2 main groups according to the American Academy of Sleep Medicine [2]: the non-OSAS group (AHI < 5) and the OSAS-group (AHI ≥ 5). The OSAS group was then distinguished into 3 sub-groups dependent on the severity of obstructive sleep apnea such as mild (5 ≤ AHI < 15), moderate (15 ≤ AHI < 30), or severe (AHI ≥ 30).

#### 2.1.3. Surgical Procedures

The indications for palatoplasty and palatopharyngoplasty were consistent with Friedman [23].

**Figure 1:**
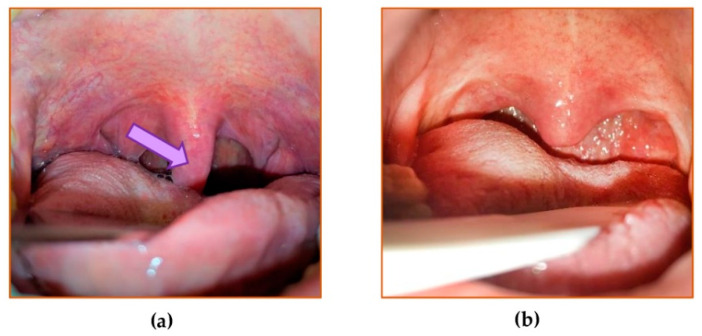
Oropharyngeal view before and after palatopharyngoplasty in a participant suffering from moderate obstructive sleep apnea syndrome (OSAS). (**a**) The view before the surgery. The elongated, thick uvula with an enlarged ovular mucosa (arrow) and webbing of the posterior pillars are shown. (**b**) The view two months after the surgery. The reduction of the mucosa of the uvula is shown.

All patients from the non-OSAS group underwent palatoplasty using radiofrequency-induced thermotherapy with ablation of uvula tissues. The procedures were performed under local anesthesia. For the study purposes, before the surgically planned ablation, a mucosal biopsy was obtained. The specimen depth included the entire submucosa, down to the level of uvula muscle, but did not include any muscle fibers (Figure 1). Only the enlarged mucosa that was planned to be removed for the treatment was included in the biopsy specimen for the study. For the patients in the OSAS-group who were selected to undergo palatopharyngoplasty under general anaesthesia, the procedure to obtain a biopsy specimen from the mucosa of the uvula was similar to the non-OSAS group. The specimen biopsy was taken within the first 15 min of all surgical procedures, irrespective of the type of the procedure. Although there was some variability between the size of biopsy specimens, they weighted a minimum of 0.05 g, which was sufficient for the study purposes. All collected specimens of uvular mucosa were evaluated macroscopically and found to be adequate to estimate the oxidative/reductive status. They were immediately frozen in −70 °C and kept for further evaluation.

### 2.2. Determination of Markers of Oxidative/Antioxidative Status

TOS and TAS were determined in the aliquots of homogenates of the uvular mucosa and expressed as mmoL/g of the wet tissue weight.

#### 2.2.1. Preparation of the Aliquots of the Uvular Mucosa

Known weight specimens of uvular mucosa were homogenized with the use of a high-performance homogenizer (Ultra-Turrax T25, IKA, Staufen, Germany) to prepare 10% (w/v) homogenates in cold potassium phosphate buffer (50 mM, pH = 7.4). The buffer was received by mixing 50 mM potassium dihydrogen phosphate (POCh, Gliwice, Poland) and 50 mM dipotassium hydrogen phosphate (POCh) prepared with the use of ultra-pure water (taken from the compact water purification system Select HP 40; Purite Ltd., Thame, Oxfordshire, UK). To avoid autooxidation, butyl-hydroxytoluene (Sigma-Aldrich Gmbh, Steinheim, Germany) in acetonitrile (Merck, Darmstadt, Germany) was added (0.01 mL of 0.5 M butyl-hydroxytoluene per 1 mL of homogenate). Homogenates prepared in this way were centrifuged (MPW-350R centrifuge, Medical Instruments, Warsaw, Poland) at 700 × *g* for 20 min at 4 °C, and the aliquots were immediately separated and stored at −70 °C until all measurements were performed.

#### 2.2.2. Total Oxidative Status (TOS) and Total Antioxidative Status (TAS) Assay

Both parameters were determined in duplicate using commercially available enzyme-linked immunosorbent assay (ELISA) kits by Immundiagnostik AG (Bensheim, Germany). The analyses were performed strictly following the producer’s instructions. TOS was measured in the aliquots of the uvular mucosa using the PerOx (TOS/TOC) Kit on the basis of the determination of total lipid peroxides present in the investigated sample in the reaction with peroxidase at 450 nm. TAS was determined using the ImAnOx (TAS/TAC) ELISA Kit on the basis of the reaction of the elimination of hydrogen peroxide added into the investigated sample by antioxidants present in the sample. The residual hydrogen peroxide generates products that absorb at 450 nm. The quantification of investigated parameters was performed with the use of ELISA universal microplate reader Epoch (BioTek Instruments, Inc; Winooski, VT, USA).

The analytical quality of these assays was checked by the measurement of particular parameters in control samples included in the kits and was conducted on the basis of the intra- and inter-assay coefficient of variation (CV). The values of TOS and TAS determined by us in the control samples included in the kits agreed with the certified values (Table S1). The intra-assay CVs were <4.5% and 3.4% for TOS and TAS, respectively, whereas the inter-assay CVs were < 5% and 2.5%, respectively. The quality control of TOS and TAS measurements confirmed the reliability of the obtained results.

#### 2.2.3. Estimation of the Extent of Oxidative Stress

The severity of oxidative stress was estimated on the basis of the value of OSI calculated as the ratio of TOS and TAS (OSI = TOS/TAS).

### 2.3. Statistical Analysis

Statistical analyses were performed using the software Statistica 13 (StatSoft, Tulsa, OK, USA). Data were first tested for normal distribution using the Shapiro–Wilk test. Since there was no normal distribution of the data, a nonparametric Mann–Whitney test was conducted for comparisons between two groups (non-OSAS group vs. OSAS group), whereas for multiple comparisons, the ANOVA signed-rank Kruskal–Wallis test was performed. Differences were considered statistically significant at *p* < 0.05. The Hedge’s *g* statistic was used to measure the effect sizes for the difference (the strength of the difference) in the values of TOS, TAS, and OSI between 2 groups (AHI ≥ 5 group, 5 ≤ AHI < 15 group, 15 ≤ AHI < 30 group or AHI ≥ 30 group vs. AHI < 5 group). Effect sizes ≥ 0.2 and < 0.5, ≥ 0.5 and < 0.8, and ≥ 0.8 are described as small, medium, and large, respectively. Moreover, to estimate mutual dependences between particular parameters, we performed Pearson correlation analysis. Correlations were considered statistically significant at the correlation coefficient (*r*) of *p* < 0.05.

## 3. Results

### 3.1. OSAS Severity

A total number of 128 individuals (6 women and 122 men; female–male ratio 1:20) between 21 and 78 years old (mean age 45.8 years) were included in the study. The characteristics of the participants of the study are presented in Table 1. BMI ranged between 21.5 and 34.9 (mean BMI 30.7). Non-smokers constituted 73% of all participants, whereas smokers (27% of participants) smoked no more than 5 cigarettes a day.

By polygraphy, 40 non-OSAS (AHI < 5), 32 mild OSAS (5 ≤ AHI < 15), 25 moderate OSAS (15 ≤ AHI < 30), and 31 severe OSAS (AHI ≥ 30) individuals were identified. The mild, moderate, and severe OSAS participants constituted 36.4%, 28.4%, and 35.2%, respectively, of all patients with OSAS (AHI ≥ 5; 88 individuals). The distribution of AHI in the particular sub-groups of participants with OSAS is presented in Table 2.

In the OSAS group, the age of individuals ranged between 26 and 78 and did not differ compared to the non-OSAS group, in which the age ranged from 21 to 73. The age in the mild, moderate, and severe sub-groups of OSAS participants was within the following ranges 26–78, 30–76, and 31–72, respectively.

In the non-OSAS group, the ESS score was 7.50 (median) and ranged from 2.00 to 11.0, while in the OSAS group it was 10.5 and ranged from 1.00 to 24.0. The ESS scores in the mild and moderate OSAS groups reached 10.5 (range: 5.00–21.0) and 9.00 (range: 1.00–15.0), respectively. The highest score of ESS, i.e., 11.0 (range: 1.00–24.0) was noted in the severe OSAS group.

VAS in the non-OSAS group was within the range of 8.0 and 10.0 and reached 9.0 (median), whereas the median value of this parameter in the OSAS group reached 9.0 and ranged from 6.0 to 10.0. The median value of VAS in the mild, moderate, and severe OSAS groups reached 9.0 (range: 7.0–10.0), 8 (range: 6.0–10.0), and 8.0 (range: 7.0–10.0), respectively.

### 3.2. Type of Surgery

Seventy-five patients (Table 3) underwent palatoplasty using radiofrequency-induced thermotherapy with the removal of enlarged uvular mucosa. The procedure was performed under local anesthesia and lasted approximately 30 min. The submucosal infiltration with 2% lidocaine (Aesculap Chifa, Nowy Tomysl, Poland) (3 mL) was used as local anesthesia. Patients selected for palatopharyngoplasty (*n* = 53; Table 3) underwent the procedure under general anesthesia which lasted 1–1.5 h. Total intravenous anesthesia was used. The following medications were administrated to perform general anesthesia: target control infusion of 1% propofol (Fresenius Kabi, Blonie, Poland), remifentanil (Aesculap Chifa, Nowy Tomysl, Poland), and cisatracurium (Accord Healthcare, Matoda, India). The total length of time to take the specimen biopsy was the same in each surgical procedure. The biopsy was performed during the first 15 min of the surgery in each case.

### 3.3. Mean Oxygen Saturation (MOS), Lowest Oxygen Saturation (LOS), and Oxygen Desaturation Index (ODI)

The level of MOS, determined as percentages, in the OSAS participants did not differ in comparison to the non-OSAS participants (Table 4). However, the value of MOS in the severe OSAS individuals was lower than in the non-OSAS group and the mild OSAS sub-group (by 2.2% and 1.9%, respectively), and tended to be lower in comparison to the moderate OSAS sub-group (Table 4). The level of LOS in the OSAS group was lower (by 7%) than in the non-OSAS group (Table 4). The values of LOS in the moderate and severe OSAS sub-groups were lower (by 8.1% and 9.3%, respectively) when compared to the non-OSAS group (Table 4). Moreover, LOSs in the moderate and severe OSAS participants were lower (by 4.8% and 6%, respectively) than in the mild OSAS participants (Table 4).

In the OSAS group, ODI was 7.7-fold higher than in the non-OSAS group (Table 4). There were no differences in ODI that were dependent on the severity of OSAS; however, this parameter in the mild, moderate, and severe OSAS sub-groups was 3.2-, 7-, and 10-fold, respectively, higher than in the non-OSAS group (Table 4).

### 3.4. Total Oxidative Status (TOS)

**Figure 2:**
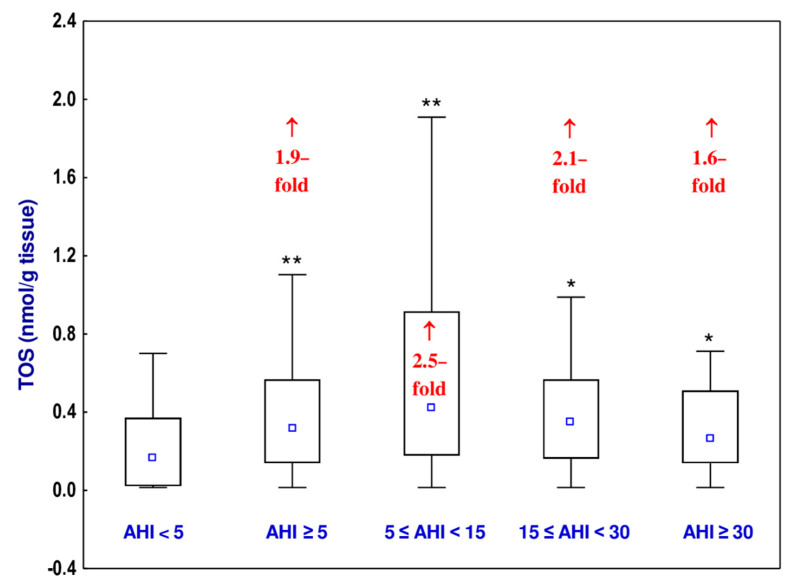
Total oxidative status (TOS) of the uvular mucosa of the participants with obstructive sleep apnea syndrome (OSAS) and in non-OSAS participants. Data represent median, 25–75% confidence interval, as well as minimum and maximum. * *p* < 0.05, ** *p* < 0.01 compared to apnea–hypopnea index (AHI) < 5. ↑—increase compared to AHI < 5.

A 1.9-fold increase in the TOS of the uvular mucosa was noted in the OSAS group as compared to the non-OSAS group (Figure 2, Table S2). The values of TOS in the mild OSAS, moderate OSAS, and severe OSAS sub-groups were higher (1.6- to 2.5-fold) than in the non-OSAS group, but there were no differences in the value of this parameter depending on the severity of OSAS (Figure 2, Table S2). The Hedge’s *g* statistic revealed small, medium, or large effect sizes for the differences in the value of TOS between the OSAS group (AHI ≥ 5) and non-OSAS group (AHI < 5), as well as between the mild (5 ≤ AHI < 15), moderate (15 ≤ AHI < 30), or severe (AHI ≥ 30) OSAS groups and the AHI < 5 group (Table 5).

### 3.5. Total Antioxidative Status (TAS)

**Figure 3:**
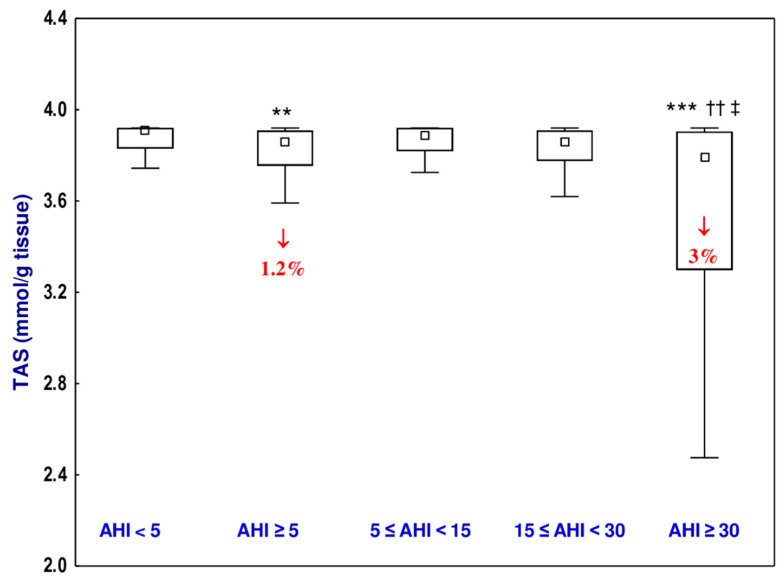
Total antioxidative status (TAS) of the uvular mucosa of the participants with obstructive sleep apnea syndrome (OSAS) and in non-OSAS participants. Data represent median, 25–75% confidence interval, as well as minimum and maximum. ** *p* < 0.01, *** *p* < 0.001 compared to apnea–hypopnea index (AHI) < 5; ^††^ *p* < 0.01 compared to 5 ≤ AHI < 15; ^‡^ *p* < 0.05 compared to 15 ≤ AHI < 30. ↓—decrease compared to AHI < 5.

The value of TAS of the uvular mucosa in the OSAS group was lower (by 1.2%) than in the non-OSAS group (Figure 3, Table S2). Moreover, TAS in the severe OSAS sub-group was lower than in the non-OSAS group (by 3%) and the mild (by 2.5%) and moderate (by 1.7%) OSAS sub-groups (Figure 3, Table S2). The Hedge’s g statistic revealed small or large effect sizes for the differences in the value of TAS between the non-OSAS group and OSAS group or severe OSAS group, respectively (Table 5).

### 3.6. Oxidative Stress Index (OSI)

**Figure 4:**
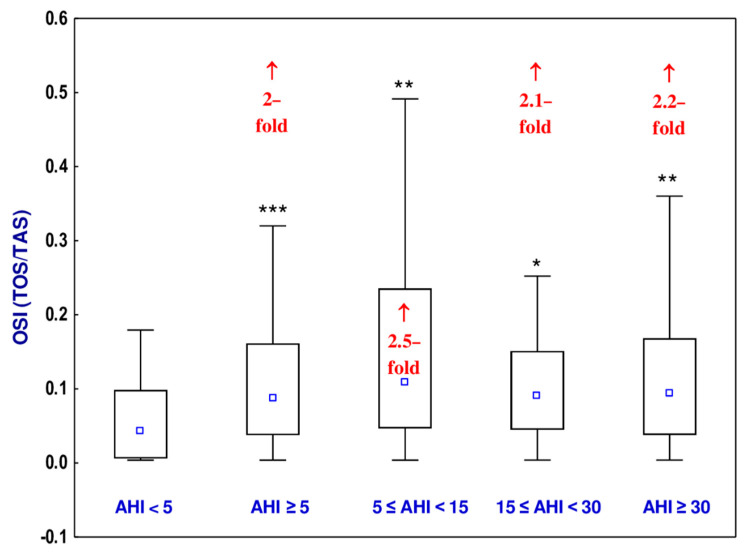
Oxidative stress index (OSI) in the uvular mucosa of the participants with obstructive sleep apnea syndrome (OSAS) and in non-OSAS participants. Data represent median, 25–75% confidence interval, as well as minimum and maximum. * *p* < 0.05, ** *p* < 0.01, *** *p* < 0.001 compared to apnea–hypopnea index (AHI) < 5. ↑—increase compared to AHI < 5.

The value of OSI of the uvular mucosa in the OSAS group, as well as in all sub-groups of the OSAS patients (mild, moderate, and severe), was higher (from 2- to 2.2-fold) than in the non-OSAS group (Figure 4, Table S2). According to the Hedge’s *g* statistic, the effect sizes for the differences in the value of OSI between the non-OSAS group and OSAS group, as well as the mild, moderate, or severe OSAS groups and the non-OSAS group, were small to large (Table 5).

### 3.7. Mutual Dependences between the Investigated Parameters

Numerous positive or negative correlations were noted between the investigated parameters (Table 6). The value of AHI, reflecting the severity of OSAS disorders, positively correlated with the age of participants and ODI and negatively correlated with TAS, MOS, and LOS. Positive correlations occurred between TOS and OSI, TOS and ODI, OSI and ODI, TAS and MOS, TAS and LOS, as well as between LOS and MOS. Moreover, there were negative correlations between TAS and ESS, and between MOS and ESS, age of the participants, and BMI. A tendency to a negative correlation between ODI and TAS and LOS was noted. A positive correlation between VAS and BMI, as well as a tendency to positive dependence between VAS and TOS or OSI, were revealed.

## 4. Discussion

The present investigation is the first one focused on the oxidative/antioxidative status in palatal tissue of sleep apnea patients and the association of oxidative stress in the uvular tissue and the presence of OSAS. The research provided evidence for disturbing a balance between oxidants and antioxidants and inducing oxidative stress in the mucosa of the uvula of patients with OSAS.

The exact relationships between pathogenic mechanisms in OSAS remain unclear. Recurrent episodes of breathing cessation during sleep expose the cardiovascular system to cycles of significant hypoxia, exaggerated negative intrathoracic pressure, and arousals. In OSAS cases, repetitive hypoxia and reoxygenation occur during sleep, inducing the oxidative stress response. The repeated hypoxia and reoxygenation cycle is similar to hypoxia-reperfusion injury, which initiates oxidative stress. Oxidative stress is considered to be part of pathophysiological changes in OSAS contributing in consequence to the neural, cardiovascular, and metabolic alterations [24]. All publications that have discussed oxidative stress in sleep apnea patients evaluated it in plasma or urine obtained from patients who suffered from this disorder [25,26,27,28,29,30]. However, the majority of these patients also suffered from concomitant diseases, i.e., hypertension, diabetes, and arrhythmia, which are related to destroying the oxidative/antioxidative balance. Our recently published systematic review showed the extent of oxidative stress associated with OSAS; however, with no clear difference between OSAS patients with and without cardiovascular complications [31]. Moreover, we found studies reported no differences in levels of oxidative stress biomarkers between groups of OSAS patients with biometrical differences (age, BMI, AHI) [31]. When this was taken into consideration, in order to better explain the relationship between oxidative stress and the severity of obstructive sleep apnea, we identified the need to investigate abnormities in the oxidative/reductive status in tissues gathered from OSAS patients during sleep surgeries, i.e., palatoplasty and palatopharyngoplasty. Decision-making for the management of OSAS is complex. It is recommended by the American Academy of Sleep Medicine guidelines and is also ethical to try appropriate non-invasive treatment options first and reserve the surgical treatment options for non-compliant patients and treatment failures. This current study did not investigate the effects of surgery on oxidative stress in the tissues. However, it could be hypothesized that both successful non-surgical and surgical treatments would reduce oxidative stress in the tissues. These tissues vibrate (mostly soft palate with the uvula) and tend to collapse in sleep-disordered breathing patients. The results of the current study show that oxidative stress occurs in the enlarged mucosa of the uvula in OSAS patients. In fact, one can ask whether the vibration of palatal mucosa might trigger the microtrauma within tissues and extend the oxidative stress. Stål and Johansson took a palatal muscle biopsy from OSAS patients during uvulopalatopharyngoplasty in patients with a long history of snoring. The authors, on the basis of enzyme-, immunohistochemical, and morphometric studies, observed an abnormal mitochondrial distribution and a reduced capillary supply within a palatal muscle regardless of the direct influence of palatal vibration during snoring; however, oxidative/antioxidative imbalance remained unclear [32]. Oxidative stress, being a state of imbalance between antioxidant defense mechanisms and the production of oxidants, might result from a decreased antioxidant capacity or an overproduction of ROS and reactive nitrogen species or both [29]. Studies demonstrated that intermittent hypoxia in OSAS resembles hypoxia/reperfusion injury mechanisms responsible for ROS overproduction [28,33]. However, these results were based on plasma evaluation. To our knowledge, no studies have demonstrated oxidative stress in palatal tissue in OSAS patients. It is critical to assess the pathology within the vibrating tissues in primary and OSAS patients because the vibration during snoring may cause inflammation of the soft palate and the intermittent hypoxia incidents during sleep may worsen the inflammatory infiltration in the soft palate. Therefore, the inflammation leads to worsened snoring and the progression of snoring may lead to OSAS. Oxidative stress activates a variety of inflammatory mediators. Clinical evidence suggests that oxidative stress and inflammation are linked to the overproduction of ROS [34]. We postulate that both inflammation and oxidative stress within the vibrating uvula in primary and OSAS patients are important components for the development of SDB. This statement is in agreement with Stål and Johansson [32].

The higher values of TOS and OSI noted in all sub-groups of the OSAS (mild, moderate, and severe) participants of the current study compared to the non-OSAS group provide evidence that the equilibrium between oxidants and antioxidants was disturbed and that OSAS is associated with the development of oxidative stress. The value of TAS was lower in the severe OSAS group in a comparison with the non-OSAS group that also shows that the more severe the disease is, the more disturbed the balance between oxidants and antioxidants. Additionally, statistically significant differences between the mild OSAS and severe OSAS groups, as well as between moderate and severe OSAS groups with the value of TAS, suggest the likely relationship of the severity of OSAS on the extent of oxidative stress.

Moreover, the 2.5-fold higher values of TOS and OSI already in the patients with 5 ≤ AHI < 15 suggest an increased amount of ROS even in the case of a mild form of OSAS. Although we noted the highest mean level of TOS in the moderate OSAS group, all three sub-groups of the OSAS patients demonstrated a significant rise in TOS and there were no differences in the value of this parameter depending on the severity of the disease. Similar results were presented by Barceló et al. [35], who indicated decreased TAS of the plasma obtained from blood taken from OSAS patients. The authors concluded that in OSAS, the impairment of protective systems for oxidative stress occurs [35].

Oxidative stress is expected in OSAS due to repeated events of hypopneas during sleep. The consecutive hypooxygenation–reoxygenation periods accompanied by recurrent apneas and hypopneas in OSAS patients induce oxidative stress and may cause endothelial dysfunction. Thus, oxidative stress not only damages endothelial cells in the peripheral vasculature but also contributes to damage to the alveolar epithelial cells. The reduced alveolar trans-epithelial exchange rate for oxygen and carbon dioxide leads to worsened hypoxemia and hypercarbia. Increased permeability of the alveolar epithelium results in the development of intra-alveolar transudate and exudate. Another mechanism of impairment of the epithelium is a decrease in vasodilation and potentiated vasoconstriction, as well as a loss of nitric oxide (NO) bioactivity in the vessel wall [36]. It can be explained by the production of ROS due to oxidative stress that reacts rapidly with NO and results in NO deficiency [37,38].

There were previous publications on the occurrence of oxidative stress in OSAS patients [24,25,26]. However, all these studies evaluated TOS, TAS, and OSI in the plasma, not in the tissue, as was the case in the current study. Our study focused on changes that occurred in the enlarged mucosa of the uvula obtained from non-OSAS and OSAS patients. We noted that OSAS is accompanied by oxidative stress in the uvular mucosa. Because there were no differences in the value of OSI between the mild, moderate, and severe OSAS sub-groups and correlations between AHI and OSI and TOS, it cannot be concluded that the extent of oxidative stress in the uvular mucosa of patients with OSAS correlates positively with the severity of the disease. However, the higher values of TOS and OSI and lower value of TAS in OSAS individuals compared to the non-OSAS ones together with the lower values of TAS in the severe OSAS sub-group than in the mild and moderate OSAS sub-groups and the negative correlation between AHI and TAS allow for the conclusion that oxidative stress in the uvular mucosa is associated with the occurrence of OSAS. It is important to underline that although the percentage changes in the value of TAS in OSAS patients (AHI ≥ 5) and severe OSAS sub-group (AHI ≥ 30) compared to the non-OSAS group (AHI < 5) reached only 1.2–3%, small or large Hedges’ *g* effect sizes were noted for this parameter.

The changes in TOS and TAS resulting in an oxidative/antioxidative imbalance trigger further tissue dysfunction and lead to impairment of protective systems for oxidative stress. Histological changes have been reported in the literature in the uvula of patients with snoring and OSAS including the diffuse hypertrophy of mucosal glands, atrophy, and interstitial fibrosis disrupting the normal tissue architecture. Moreover, the extensive edema of the lamina propria of the soft palate vessels with vascular congestions and dilatation was observed [39]. Sekosan et al. observed plasma cell infiltration and interstitial edema with the uvula removed during uvulopalatopharyngoplasty in OSAS patients [40]. Another group of researchers obtained enlarged mucosa of the uvula also during sleep surgery. The authors performed the immunohistochemical analysis and revealed structural changes caused by the vibration of these tissues during snoring. It may participate in the upper away collapsibility [41]. A local inflammatory process (significant inflammatory infiltration and edema) within soft palate tissues in the OSAS patient was also confirmed by us on the basis of hematoxylin and eosin staining of a specimen obtained from the hypertrophic mucosa of the uvula (Figure S1).

We are aware of the limitations of this study. The most important is the evaluation of oxidative/reductive status only in the uvula and on the basis of the determination of only TOS and TAS. This was due to the fact that only the enlarged mucosa of the uvula was surgically removed. Because in some cases the enlarged mucosa of the uvula was very small, it was not enough to determine other indices of the oxidative/reductive status. Although we believe that this is acceptable for the current study, in the future, additional samples from other regional tissues, when feasible and ethical, should be considered for a more comprehensive histopathological assessment of upper airway tissues in order to provide stronger evidence on pathogenetic mechanisms of OSAS or oxidative stress in this disease. However, TOS and TAS, together with OSI, are the most reliable parameters to reflect destroying the oxidative/antioxidative balance. Moreover, the total length of time of the surgery was different among study groups; however, the time to obtain specimen biopsy was the same in each case. The surgery and the anesthesia itself may potentially cause oxidative stress; however, only 15 min was required to take the specimen biopsy. According to the literature, local and general anesthesia that lasts less than 2 h should not induce oxidative stress [42,43]. Therefore, it can be assumed that the anesthesia that the patients underwent might have no impact on the oxidative/reductive balance of the uvula. Another limitation is the sleep study type III, performed during the diagnostic process. We are aware of the limitation of this type of sleep study in identifying arousals, in quantifying AHI, and in its inability to assess the sleep structure. However, participants were given the device home for two nights, which is beneficial compared to one-night polysomnography (PSG). A first night effect of the sleep study is seldomly observed. The participants enrolled in our study were thoroughly instructed how to use the device and how to put it on. We excluded individuals with comorbidities from the study. Moreover, the American Academy of Sleep Apnea allows for performing a sleep study type III when the patient is free from significant comorbid conditions and for a preoperative clinical evaluation [2,44]. Studies showed that a home portable monitoring device demonstrated a high level of diagnostic agreement with a simultaneous PSG and performed valid home diagnostic studies for OSAS, especially when manually scored. The authors demonstrated that the difference between the AHI from the reference PSG and the home study was similar to the difference between the PSGs [45]. Bibbins-Domingo et al. also show home sleep study as a viable alternative to PSG in selected circumstances and recommend it for diagnosis of OSAS in uncomplicated patients with an increased risk of moderate-to-severe OSAS [46]. The sleep study was manually read by the first author of the current study. At this stage of our research, it is difficult to determine the cause–effect relationship between OSAS and oxidative stress. OSAS could be either the cause of oxidative stress or destroying of the oxidative antioxidative balance may be involved in the development and occurrence of OSAS.**** Exclusion of patients with severe obesity does not imply that we do not think that oxidative stress is not associated with their OSAS; on the contrary, we anticipate that it would be. However, because oxidative stress may be present in the tissues just because of the presence of regional excessive fat accumulation, we would not be able to claim that these results are directly associated only with the presence and severity of OSAS. We, therefore, selected inclusion criteria that would more likely demonstrate the association without the confounding factor of obesity. Moreover, due to broader contraindications for surgical treatment in severely obese patients, we would not have enough such patients to perform subgroup analysis to differentiate this effect. In the future, trials with larger sample size and broader inclusion criteria may show this association in obese patients also.

Several studies demonstrate new treatment protocols for primary snoring and OSAS patients including nonsurgical and surgical procedures [1,21,22]. A variety of different devices, such as oral appliances, vests, positional therapy, and positive airway pressure therapy, may be ineffective with poor compliance, as well as cause back pain and discomfort during sleep. Sleep surgery has a risk of complications and failures. Therefore, searching for alternative treatment in SDB is needed. Some studies showed a change in TAS with the treatment of OSAS. After 12 months of positive airway pressure therapy, TAS normalized [34]. Effective positive airway pressure therapy recommended for OSAS patients led to the improvement of antioxidant defense and diminish oxidative stress-related markers [34,35,47]. This sheds new light on the future directions of research to find effective therapies for SDB patients. 

## 5. Conclusions

The present study provides new and important data on destroying the oxidative/antioxidative balance and the evidence of oxidative stress in the palatal tissue in OSAS patients. On the basis of the findings, we can conclude that oxidative stress of the uvular mucosa is associated with the occurrence of OSAS among adults. More studies are necessary to further clarify the cause of tissue oxidative stress in OSAS patients and its association with factors such as obesity, smoking, hypertension, diabetes, and dyslipidemia. 

## Figures and Tables

**Table 1:** Age and body mass index (BMI) of the male and female participants of the study.

Group	Parameter	Male		Female	
		Median	Min.–Max.	Median	Min.–Max.
Non-OSAS (AHI < 5); *n* = 40	age	38.0	21.0–73.0	53.0	51.0–55.0
Male = 38; Female = 2	BMI	29.9	29.0–34.8	27.1	21.5–32.7
OSAS participants (AHI ≥ 5); *n* = 88	age	46.0	26.0–78.0	44.5	32.0–61.0
Male = 84; Female = 4	BMI	30.9	25.1–34.9	30.8	29.4–34.6

OSAS, obstructive sleep apnea syndrome; AHI, apnea–hypopnea index; *n*, number of individuals; Min.–Max., minimum–maximum.

**Table 2:** The apnea–hypopnea index (AHI) in the participants with obstructive sleep apnea syndrome (OSAS) and in non-OSAS participants.

Group	*n*	AHI	
		Median	Min.–Max.
Non-OSAS (AHI < 5)	40	4.00	0.10–4.90
OSAS participants (AHI ≥ 5)	88	20.75 ***	5.30–70.6
Mild OSAS (5 ≤ AHI < 15)	32	11.25 ***	5.30–14.5
Moderate OSAS (15 ≤ AHI < 30)	25	21.00 ***^,†††^	15.2–29.7
Severe OSAS (AHI ≥ 30)	31	46.00 ***^,†††,‡‡‡^	30.1–70.6

*n*, number of individuals; Min.–Max., minimum–maximum; *** *p* < 0.001 compared to AHI < 5; ^†††^ *p* < 0.01 compared to 5 ≤ AHI < 15; ^‡‡‡^ *p* < 0.05 compared to 15 ≤ AHI < 30.

**Table 3:** Type of the surgery performed in the participants with obstructive sleep apnea syndrome (OSAS) and in non-OSAS participants.

Group	Total Number of Participants Subjected to Surgery	Number of Participants Subjected to Surgery under Local Anesthesia (% of All Patients)	Number of Participants Subjected to Surgery under General Anesthesia (% of All Patients)
Non-OSAS (AHI < 5)	40	40 (100%)	0
OSAS participants (AHI ≥ 5)	88	35 (40%)	53 (60%)
Mild OSAS (5 ≤ AHI < 15)	32	22 (69%)	10 (31%)
Moderate OSAS (15 ≤ AHI < 30)	25	9 (36%)	16 (64%)
Severe OSAS (AHI ≥ 30)	31	4 (13%)	27 (87%)

AHI, apnea–hypopnea index.

**Table 4:** Oxygen saturation level (MOS), lowest oxygen saturation level (LOS), and oxygen desaturation index (ODI) in the participants with obstructive sleep apnea syndrome (OSAS) and in non-OSAS participants.

Group	*n*	MOS [%] Median (Min.–Max.)	LOS [%] Median (Min.–Max.)	ODI [%] Median (Min.–Max.)
Non-OSAS (AHI < 5)	40	94.10 (85.3–96.0)	86.00 (79.0–94.0)	4.0 (0.5–5.0)
OSAS participants (AHI ≥ 5)	8	93.00 (77.0–96.0)	80.00 * (50.0–91.0)	30.8 *** (10.1–67.8)
Mild OSAS (5 ≤ AHI < 15)	32	93.80 (90.1–96.0)	83.00 (69.0–91.0)	12.8 * (10.1–20.0)
Moderate OSAS (15 ≤ AHI < 30)	25	93.05 (88.0–96.0)	79.00 **^,††^ (50.0–84.0)	27.8 *** (23.4–37.9)
Severe OSAS (AHI ≥ 30)	31	92.00 *^,††,#^ (77.0–96.0)	78.00 **^,††^ (51.0–89.0)	40.1 *** (24.3–67.8)

AHI, apnea–hypopnea index; *n*, number of individuals; Min.–Max., minimum–maximum; * *p* < 0.05, ** *p* < 0.01, *** *p* < 0.001 compared to AHI < 5; ^††^ *p* < 0.01 compared to 5 ≤ AHI < 15; ^#^ *p* = 0.05 compared to 15 ≤ AHI < 30.

**Table 5:** Hedge’s *g* measurement of the effect sizes for the difference in the values of total oxidative status (TOS), total antioxidative status (TAS), and oxidative stress index (OSI).

Group	*n*	Hedges’ *g*		
		TOS	TAS	OSI
**OSAS participants (AHI ≥ 5)**	88	0.430 ***	0.349 ***	0.436 ***
**Mild OSAS (5 ≤ AHI < 15)**	32	0.840 ***	0.016 ^NS^	0.840 ***
**Moderate OSAS (15 ≤ AHI < 30)**	25	0.655 ***	0.052 ^NS^	0.640 ***
**Severe OSAS (AHI ≥ 30)**	31	0.435 ***	0.829 ***	0.543 ***

*n*, number of individuals; AHI, apnea–hypopnea index. Effect sizes ≥ 0.2 and <0.5, ≥0.5 and <0.8, and ≥0.8 are described as small, medium, and large, respectively. *** *p* < 0.001 compared to AHI < 5. ^NS^ *p* > 0.05 (not statistically significant).

**Table 6:** Mutual dependences between the investigated parameters.

Variable	Age	BMI	AHI	ESS	VAS	MOS	LOS	ODI	TOS	TAS
BMI	NS	–								
AHI	0.207 *	NS	–							
ESS	NS	NS	NS	–						
VAS	NS	0.527 ^‡^	NS	NS	–					
MOS	–0.279 *	–0.378 *	–0.366 ^‡^	–0.310 *	NS	–				
LOS	NS	NS	–0.366 ^†^	NS	NS	0.584 ^‡^	–			
ODI	NS	NS	0.932 ^‡^	NS	NS	NS	–0.428 ^#^	–		
TOS	NS	NS	NS	NS	0.266 ^#^	NS	NS	0.342 ^†^	–	
TAS	NS	NS	–0.286 ^†^	–0.343 *	NS	0.515 ^‡^	0.259 *	–0.220 ^#^	NS	–
OSI	NS	NS	NS	NS	0.267 ^#^	NS	NS	0.395 ^‡^	0.982 ^‡^	NS

The results of Pearson correlation analysis are expressed as *r* values and the level of statistical significance (*p*). The values of *r* with *p* < 0.05 were considered statistically significant (* *p* < 0.05, ^†^ *p* < 0.01, ^‡^ *p* < 0.001, ^#^ *p =* 0.06–0.07). NS—not statistically significant (*p* > 0.05). BMI, body mass index; AHI, apnea–hypopnea index; ESS, Epworth Sleep Scale; VAS, a visual analog scale for snoring; MOS, mean oxygen saturation; LOS, lowest oxygen saturation; ODI, oxygen desaturation index; TOS, total oxidative status; TAS, total antioxidative status; OSI, oxidative stress index.

## Data Availability

The data presented in this study are available on request from the corresponding author. The data are not publicly available.

## Supplementary Materials

The following are available online at https://www.mdpi.com/2077-0383/10/5/1132/s1: Figure S1: Uvular mucosa obtained from an individual suffered from obstructive sleep apnea syndrome (OSAS). Table S1: Analytical quality of total oxidative status (TOS) and total antioxidative status (TAS) measurements in certified reference samples. Table S2: Total oxidative status (TOS), total antioxidative status (TAS), and oxidative stress index (OSI) in the uvular mucosa of the participants with obstructive sleep apnea syndrome (OSAS) and in non-OSAS participants.

## Abbreviations

AHI	apnea–hypopnea index
BMI	body mass index
CD	cluster of differentiation
CV	coefficient of variation
ELISA	enzyme-linked immunosorbent assay
ESS	Epworth Sleep Scale
LOS	lowest oxygen saturation
MOS	mean oxygen saturation
NO	nitric oxide
ODI	oxygen desaturation index
OSAS	obstructive sleep apnea syndrome
OSI	oxidative stress index
*p*	level of statistical significance
PSG	polysomnography
*r*	correlation coefficient
ROS	reactive oxygen species
SDB	sleep-disordered breathing
TAS	total antioxidative status
TOS	total oxidative status
VAS	visual analogue scale for snoring
