# Supplementary material for: The Association of Oxidative Stress in the Uvular Mucosa with Obstructive Sleep Apnea Syndrome: A Clinical Study
Source: J Clin Med. 2021 Mar 8;10(5):1132. doi: 10.3390/jcm10051132 (PMC7962821; doi:10.3390/jcm10051132)
Supplement: Supplementary file 1 [file jcm-10-01132-s001.pdf]

Journal of
Clinical Medicine

MDPI

Supplementary Materials

Table S1. Analytical quality of total oxidative status (TOS) and total antioxidative status (TAS) measurements in certified reference samples.

Number of Kit		Reference Values [µmol/L]	Noticed Values [A] [µmol/L]	Precision (CV) [B]
**PerOx (TOS/TOC) Kit**				
**I**	Sample 1	162–220	174 ± 6.45	3.1%
	Sample 2	204–276	236 ± 6.00	
**II**	Sample 1	162–220	177 ± 6.00	2.9%
	Sample 2	204–276	236 ± 5.50	
**III**	Sample 1	162–220	200 ± 5.00	1.9%
	Sample 2	204–276	260 ± 3.50	
**IV**	Sample 1	170–230	200 ± 6.50	3.4%
	Sample 2	195–263	230 ± 8.00	
**ImAnOx (TAS/TAC) ELISA Kit**				
**I**	Sample 1	169–282	229 ± 4.60	3.0%
	Sample 2	407–678	495 ± 13.0	
**II**	Sample 1	170–283	175 ± 7.00	3.6%
	Sample 2	437–728	493 ± 16.0	
**III**	Sample 1	185–308	195 ± 8.00	4.5%
	Sample 2	385–641	416 ± 21.0	
**IV**	Sample 1	185–308	190 ± 4.50	1.7%
	Sample 2	385–641	420 ± 4.50	

[A] Data are represented as mean ± standard deviation (SD) for two measurements. [B] Precision of measurements is expressed as a coefficient of variation (CV).

Table S2. Total oxidative status (TOS), total antioxidative status (TAS), and oxidative stress index (OSI) in the uvular mucosa of the participants with Obstructive Sleep Apnea Syndrome (OSAS) and non-OSAS participants.

Group	n	TOS [mmol/g tissue] Median (Min.–Max.)	TAS [mmol/g tissue] Median (Min.–Max.)	OSI Median (Min.–Max.)
Non-OSAS (AHI < 5)	40	0.168	3.908	0.043
		(0.0150–1.0290)	(3.1640–3.920)	(0.0038–0.2874)
OSAS participants (AHI ≥ 5)	88	0.314 **	3.860 **	0.087 ***
		(0.0150–7.3690)	(1.9540–3.920)	(0.0038–1.8800)
Mild OSAS (5 ≤ AHI < 15)	32	0.422 **	3.888	0.109 **
		(0.0150–2.4863)	(3.320–3.920)	(0.0038–0.6373)
Moderate OSAS (15 ≤ AHI < 30)	25	0.353 *	3.858	0.090 *
		(0.0150–6.8750)	(3.2960–3.920)	(0.0040–1.8250)
Severe OSAS (AHI ≥ 30)	31	0.262 *	3.792 *** †† ‡	0.094 **
		(0.0150–7.3690)	(1.9540–3.920)	(0.0039–1.8800)

n, number of individuals; Min.–Max., minimum–maximum; AHI, apnea/hypopnea index; *p < 0.05, **p < 0.01, ***p < 0.01 compared to AHI < 5; ††p < 0.01 compared to 5 ≤ AHI < 15; ‡p < 0.05 compared to 15 ≤ AHI < 30.

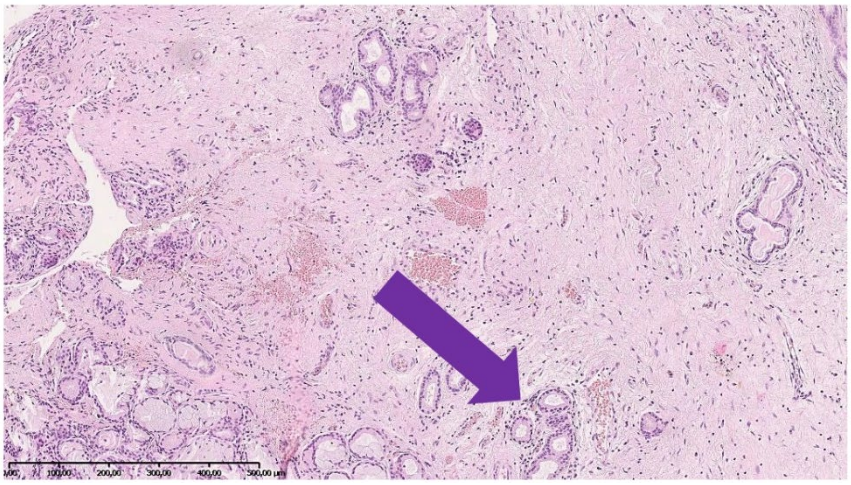

Figure S1. Uvular mucosa obtained from an individual suffered from Obstructive Sleep Apnea Syndrome (OSAS). Haemotoxylin and eosin (H+E) staining showing significant inflammatory infiltration and edema (arrow). Magnification ×200.
